# On estimation of genetic variance within families using genome-wide identity-by-descent sharing

**DOI:** 10.1186/1297-9686-45-32

**Published:** 2013-09-03

**Authors:** William G Hill

**Affiliations:** 1 Institute of Evolutionary Biology, School of Biological Sciences, University of Edinburgh, West Mains Road, Edinburgh EH9 3JT, UK

## Abstract

**Background:**

Traditionally, heritability and other genetic parameters are estimated from between-family variation. With the advent of dense genotyping, it is now possible to compute the proportion of the genome that is shared by pairs of sibs and thus undertake the estimation within families, thereby avoiding environmental covariances of family members. Formulae for the sampling variance of estimates have been derived previously for families with two sibs, which are relevant for humans, but sampling errors are large. In livestock and plants much larger families can be obtained, and simulation has shown sampling variances are then much smaller.

**Methods:**

Based on the assumptions that realised relationship of sibs can be obtained from genomic data and that data are analyzed by restricted maximum likelihood, formulae were derived for the sampling variance of the estimates of genetic variance for arbitrary family sizes. The analysis used statistical differentiation, assuming the variance of relationships is small.

**Results:**

The variance of the estimate of the additive genetic variance was approximately proportional to 1/ (*fn*^2^$\sigma_{R}^{2}$), for *f* families of size *n* and variance of relationships $\sigma_{R}^{2}$.

**Conclusions:**

Because the standard error of the estimate of heritability decreased in proportion to family size, the use of within-family information becomes increasingly efficient as the family size increases. There are however, limitations, such as near complete confounding of additive and dominance variances in full sib families.

## Background

Quantitative genetic parameters such as heritability have traditionally been estimated from the variation among full- or half-sib families, or from the parent-offspring covariance [1,2]. The covariance among sibs is assumed to be proportional to the pedigree relationship, but relatives may be further correlated because they share a common environment. This problem arises particularly in humans and, although sire families can be used in livestock to minimise the environmental covariance of sibs, these and weaker relationships come at the cost of higher sampling errors of heritability estimates because the correlation between sibs has to be multiplied by the inverse of the relationship to obtain an estimate of heritability. Estimates of heritability from non-pedigreed populations also rely heavily on getting good estimates of pedigree relationship [3], which is difficult unless relationships are very close, and environmental confounding can still a source of bias.

Although pairs of full-sibs, for example, share half their genome on average, individual pairs do not because of Mendelian sampling of large chromosome segments. Such a discrepancy at pairs of loci is the basis of QTL (quantitative trait locus) mapping using, for example, the method of Haseman and Elston [4], to associate the phenotypic divergence between sibs to differences in marker frequency. Dense marker genomes are now available, and Visscher et al. [5] proposed that the actual or realised relationships between sibs can be estimated from genomic data and the association between the actual relationship and phenotypic similarity used to estimate the genetic covariance within families, thereby eliminating correlations due to shared environment. Visscher and colleagues used data on human dizygotic twins and full-sibs, first from microsatellites [5] and subsequently from SNPs (single nucleotide polymorphisms) [6] to estimate the level of genome sharing and thus trait heritability. In a later paper, Visscher [7] discussed the theory further. However, the sampling error of the estimates of genetic variance was high because the variation in actual relationship was small (typical standard deviation (SD) of 3.9% of the mean of 50% for human full-sibs, as expected from theory [5,7-10]). Since family sizes in humans are also very small, many are needed for precise estimation.

Ødegård and Meuwissen [11] pointed out that the method of Visscher et al. [5] could be used in very large families, such as for fish species, and for which it is not always practical to avoid rearing full-sibs together. They showed by simulation that sampling errors of the resulting estimates of heritability are substantially reduced as family size increases and are smaller with a few large families than with many small families. These results raise the following basic question: for a family of *n* sibs, is the information content, i.e. the inverse of the sampling variance of the estimate of heritability, approximately proportional to family size *n* (or e.g. to *n* -1) or to the number of pairs in the family, ½*n*(*n* -1)? The simulation results of Ødegård and Meuwissen [11] indicated the latter. Furthermore, PM Visscher (personal communication) showed that, using genomic relationships estimated from a sample of *N* individuals from the population, the sampling variance is a function of *N*^2^. The difference between methods with sampling variances that depend on approximately squares of numbers rather than numbers of individuals is not trivial and clearly has an important impact on their design and potential utility.

The model used by Ødegård and Meuwissen [11] was based on a finite number (80) of genomic blocks that were individually marked, and with trait effects that were identically normally distributed for each block. In this note, we quantify these estimates and show how they depend on the design and variation in realised relationships. We adopt a model in which the realised relationship is continuous over the genome and with trait effects that are uniformly distributed across the genome. To calculate sampling errors, Visscher et al. [5] used regression of the squared phenotypic difference of sibs on the estimated actual relationship from tracking genome segments, whereas Ødegård and Meuwissen [11] used a REML (restricted maximum likelihood) analysis within and between families with estimated realised relationships for a finite number of genome segments. In the present analysis the data were assumed to be analysed by REML. Implications for design of experiments are discussed.

## Analysis

Let us assume that the data are from matings of unrelated individuals and comprise *f* (≥ 1) families each of size *n* (≥ 2). The extension to variable *n* is straightforward and deferred meanwhile. The mean (i.e. pedigree) numerator relationship within families is *A* (e.g. 0.25 for half-sibs or 0.5 for full-sibs) and the within-family variance of actual relationships is $\sigma_{R}^{2}$. We also assume that all sibs share the same environment and, for simplicity, as in the work of Visscher et al. [5,6], that additive genetic variance is estimated using only within-family differences; in essence, family effects are regarded as fixed. Therefore information is accumulated independently across families and no bias or sampling error arises due to common environment, albeit at the cost of losing potential between-family genetic information.

### Additive model

Initially, we assumed that gene effects were additive but subsequently extended the results to include dominance. The additive genetic variance is $\sigma_{A}^{2}$, the residual environmental variance is $\sigma_{E}^{2}$, and so the within-family variance is $\sigma_{W}^{2}$ = (1 - *A*)$\sigma_{A}^{2}$ + $\sigma_{E}^{2}$. The phenotypic variance is given by $\sigma_{P}^{2}$ = *A*$\sigma_{A}^{2}$ + $\sigma_{C}^{2}$ + $\sigma_{W}^{2}$, where $\sigma_{C}^{2}$ is the variance due to common environment. In the analysis, it is convenient to parameterise the actual relationship between family members *i* and *j* in terms of deviations from mean pedigree-based relationships: *r*_*ij*_ = *A*_*ij*_ - *A*. The *n* × *n* covariance matrix **V** of observations **y** within a family of *n* sibs is then $\text{var}\left(y\right)=V=I\sigma_{W}^{2}+R\sigma_{A}^{2}$, where **I** is the identity matrix and elements of **R** are *r*_*ij*_, *i ≠ j*, and *r*_*ii*_ = 0.

The sampling variance of the parameter estimates can be approximated by using a Taylor series expansion in *r*_*ij*_ because these deviations are small, and then taking expectations so as to obtain Fisher’s information matrix **S** (the inverse of the variance covariance matrix) for the REML estimates of variance components ${\hat{\sigma }}_{A}^{2}$ and ${\hat{\sigma }}_{W}^{2}$, respectively. The derivation is rather complicated, so details are given in Appendix 1. For a family of size *n* it is shown that:

(1) $$S=\frac{n-1}{2\sigma_{W}^{4}}\left(\begin{array}{cc} m\sigma_{R}^{2} & -2m\sigma_{R}^{2}\sigma_{A}^{2}/\sigma_{W}^{2}\\ -2m\sigma_{R}^{2}\sigma_{A}^{2}/\sigma_{W}^{2} & 1+3m\sigma_{R}^{2}\sigma_{A}^{4}/\sigma_{W}^{4} \end{array}\right)$$

where *m* = *n*(1 – 2/*n* + 2*/n*^2^). Since between-family relationships are not used, information **S**_*k*_ from family *k* is merely summed over families, with corresponding elements for family size *n*_*k*_ and *m*_*k*_, *k* = 1, … , *f*. The overall variance-covariance matrix of the estimates is:

$$C=\left(\begin{array}{cc} \text{var}\left({\hat{\sigma }}_{A}^{2}\right) & \text{cov}\left({\hat{\sigma }}_{A}^{2},{\hat{\sigma }}_{W}^{2}\right)\\ \text{cov}\left({\hat{\sigma }}_{A}^{2},{\hat{\sigma }}_{W}^{2}\right) & \text{var}\left({\hat{\sigma }}_{W}^{2}\right) \end{array}\right)={\left(\sum_{k}S_{k}\right)}^{-1}$$

With *f* families of equal size, from (1):

(2) $$\begin{array}{ll} C= & \left(\frac{{2\sigma }_{W}^{4}}{f\left(n-1\right){m\sigma }_{R}^{2}\left({1-m\sigma }_{R}^{2}\sigma_{A}^{4}/\sigma_{W}^{4}\right)}\right)\\ & \times \left(\begin{array}{cc} 1+3{m\sigma }_{R}^{2}\sigma_{A}^{4}/\sigma_{W}^{4} & 2m\sigma_{R}^{2}\sigma_{A}^{2}/\sigma_{W}^{2}\\ 2m\sigma_{R}^{2}\sigma_{A}^{2}/\sigma_{W}^{2} & {m\sigma }_{R}^{2} \end{array}\right) \end{array}$$

The estimate of the environmental variance is ${\overset{⌢}{\sigma }}_{E}^{2}={\overset{⌢}{\sigma }}_{W}^{2}-\frac{1}{2}{\overset{⌢}{\sigma }}_{A}^{2}$ and hence $\text{var}\left({\overset{⌢}{\sigma }}_{E}^{2}\right)=c_{22}–c_{12}+\frac{1}{4}c_{11}$ and $\text{cov}\left({\hat{\sigma }}_{A}^{2},{\hat{\sigma }}_{E}^{2}\right)=c_{12}–\frac{1}{2}c_{11}$, where *c*_*ij*_ are elements of **C**. Taking just $\sigma_{A}^{2}$ and $\sigma_{E}^{2}$ into account, ${\hat{\sigma }}_{P}^{2}={\hat{\sigma }}_{A}^{2}+{\hat{\sigma }}_{W}^{2}$, and the sampling error of the corresponding heritability estimate, ${\hat{h}}^{2}={\hat{\sigma }}_{A}^{2}/{\hat{\sigma }}_{P}^{2}$, can be approximated using standard formulae for ratios (see e.g. page 818 in [2]). Between-family information, not included in the data used above, has to be incorporated to estimate the phenotypic variance and heritability if common family environment or allowance for non-additive effects is to be included.

If the quantity $m\sigma_{R}^{2}\sigma_{A}^{4}/\sigma_{W}^{4}$ is small, the determinant of **S** is dominated by its diagonal elements and var (${\overset{⌢}{\sigma }}_{A}^{2}$) simplifies to:

(3) $$\text{var}\;\left({\hat{\sigma }}_{A}^{2}\right)\approx 1/s_{11}=2\sigma_{W}^{4}/\left[f\left(n-1\right)m\sigma_{R}^{2}\right]$$

Hence for families of *n* = 2 individuals, *m* = 1 and $\text{var}\left({\hat{\sigma }}_{A}^{2}\right)\approx 2\sigma_{W}^{4}/\left(f\sigma_{R}^{2}\right)$. This corresponds to the formula of Visscher et al. [5] for the sampling error of the heritability estimate: ${2\left(1-t\right)}^{2}/\left(f\sigma_{R}^{2}\right)$, where *t* is the intra-class correlation of family members. As *n* increases, *m*(*n -* 1) = *n*(*n* – 3 + 2*/n* - 2/*n*^2^) → *n*(*n* – 3) → *n*^2^. If $\sigma_{R}^{2}$ is small and *n* large, then var(${\hat{\sigma }}_{A}^{2}$) $\sim 2\sigma_{W}^{4}/\left({fn}^{2}\sigma_{R}^{2}\right)$.

The variation in relationships within a family depends on whether family members are full- or half-sibs, on the total map length (*L*) of the chromosomes and, to a limited extent, on their individual lengths [5,7,10]. To a good approximation, $\sigma_{R}^{2}$ ~ 1/(16 *L*) – 1/(3 *L*^2^) for full-sibs and one-half of that for half-sibs [5,7]. For humans, the number of autosomes is 22 and the total map length is 35.9 M, so $\sigma_{R}^{2}$ is approximately 0.00153 for full-sibs and 0.00077 for half-sibs (SD = 0.039 and 0.028). Therefore, for full-sib families of a species with a map length and chromosome number similar to humans, SE(${\hat{\sigma }}_{A}^{2}$) ~ 36 $\sigma_{W}^{2}/\left[\sqrt{\mathrm{fn}\left(n-3\right)}\right)$, e.g. 0.28 $\sigma_{W}^{2}$ for 50 families of size 20 and 0.17 $\sigma_{W}^{2}$ for 20 families of size 50. Cattle, for example, have 29 autosomes and a map length of 32.5 M [12], so $\sigma_{R}^{2}$ would be a little larger and the sampling variance of estimates of heritability correspondingly smaller.

### Simulation check on approximations

In the analysis in Appendix 1, many simplifying assumptions were made in the Taylor series analysis. As a partial check, simulation was undertaken for a model of 22 chromosomes, each 1.632 M long, i.e. the mean length of human chromosomes, and relationships were simulated with the programme used previously to check formulae for variance in relationships [10]. (The distribution of relationships would be little affected if map lengths varied [10]). The information matrix **S** was then computed directly from equation (A1) and from the approximation in Equation (1). For simplicity, however, it was assumed that the contrast matrix **K** (see below equation (A1)) was invariant (see examples in Table 1). In general, there was good agreement between the observed and the approximate predicted estimates of sampling variance (Table 1), but this deteriorated as family size increased, with the approximation generally underestimating the sampling variance. This bias would be greater if $\sigma_{R}^{2}$ were higher. Although, if only a single chromosome was fitted $\sigma_{R}^{2}$ would be much greater, the additive variance contributed by it would be only a fraction of the total and, as the example in Table 1 shows, the approximation remains good. Table 1 also gives predictions based solely on Equation (3), showing a good fit with those obtained directly from Equation (2).

**Table 1:** **Comparison of var(**${\hat{\sigma }}_{A}^{2}$) predicted from the information matrix directly and from the Taylor series approximation^*^

**Family**	**HS**			**FS**			**FS**			**FS**			**FS**		
*h*^2^	0.5			0.25			0.5			0.75			0.04		
chr	22			22			22			22			1		
*n*	5	15	25	5	15	25	5	15	25	5	15	25	5	15	25
Eq (A1)	174	12.2	4.15	94	6.67	2.26	82.6	5.94	2.04	71.4	5.20	1.81	4.80	0.354	0.127
Eq (1)	182	11.8	3.88	101	6.56	2.18	88.6	5.83	1.97	77.1	5.23	1.82	5.26	0.331	0.110
Eq (3)	182	11.7	3.80	101	6.53	2.16	88.1	5.69	1.88	76.0	4.90	1.62	5.26	0.330	0.110

*Predictions were obtained directly by inverting the realised information matrix (eq A1) obtained from sampling relationships, and from the Taylor series approximation eq. (1) using the variance of relationships directly; variances were computed by averaging information over samples of 100 families, but are expressed for a single family, so for *f* families var(${\hat{\sigma }}_{A}^{2}$) should be divided by *f*; predictions using the simplification eq. (3) are shown similarly; results are for half (HS) and full (FS) sib families; *h*^2^ is the proportion of variance contributed by the fitted chromosomes; chr is the number of chromosomes; chr = 22 denotes the whole genome; chr = 1 denotes a single chromosome.

### Dominance

In full-sib families, both additive and dominance variance can, in principle, be estimated. Derivation of the extended information matrix is given in Appendix 2. It depends on the variance $\sigma_{Q}^{2}$ in dominance relationships (about its mean of ¼) and the covariance between dominance and additive relationships, *cov*_RQ_. However, as Visscher et al. [5] pointed out, the additive and dominance relationships within families are very highly correlated, since the additive coefficient depends on the average number of paternal and maternal genes that are shared identical by descent at a locus and the dominance coefficient on whether both are shared. The regression of dominance on additive relationships (*cov*_RQ_ / $\sigma_{R}^{2}$) is equal to 1 and their correlation is approximately 0.9. This implies that, in practice, partitioning $\sigma_{A}^{2}$ and $\sigma_{D}^{2}$ using within-family information is probably not feasible and furthermore that if only an additive model is used, the estimate of $\sigma_{A}^{2}$ is biased upwards by $\sigma_{D}^{2}$; indeed it essentially has expectation $\sigma_{A}^{2}+\sigma_{D}^{2}$.

## Discussion and conclusions

The analysis shows that the sampling variances of estimates of heritability based on within-family realized relationships fall roughly in proportion to *n*^2^ as family size *n* increases, i.e. based on the number of pairwise comparisons among individuals in the family, and in proportion to the number of families. Therefore, when undertaking such an analysis, it is more efficient to use few very large families, although one might be reluctant to use just one or very few families in case they are atypical [11]. Here, a model of a continuous genome was used, rather than a finite number of independent regions as by Ødegård and Meuwissen [11], and the calculations assumed a fairly even distribution of genetic variance along the genome. If there is much heterogeneity, e.g. a few QTL of large effect, the sampling errors of genetic variance estimates would increase. In the present analysis, we make the assumption that shared segments are identified accurately, for example using Merlin [13].

Ødegård and Meuwissen [11] investigated the effect of selectively genotyping only the individuals with high and low phenotypes within a family, when all phenotypes are included in the REML analysis. The efficiency of this approach was good in terms of sampling errors but estimates of heritability were biased downwards when sample sizes were small. This may reflect insufficient marker coverage of the genes of interest because of lack of linkage disequilibrium, in which case this bias may be hard to avoid, but possibly also bias caused by selection.

They also estimated actual relationships from a finite number of markers and, occasionally, obtained a singular matrix in their simulated replicates [11]. To check the causes, simulated relationships were sampled from a continuous chromosome model [10] and the exact allele sharing was computed. Pairs of individuals can inherit identical non-recombinant short chromosomes, thereby yielding a positive semi-definite relationship matrix (i.e. including zero but not negative eigenvalues). In the unlikely event that this occurs at all chromosomes, the data can still be analysed by REML. Negative eigenvalues were not obtained in our simulations and indeed seem infeasible, because the relationships were jointly sampled. Negative eigenvalues are a consequence of the estimation of weak relationships from marker data and might arise in practice.

A different approach to estimating the genetic variance free of common environment was suggested by Yang et al. [14]. They fitted by regression all the SNPs to data from individuals sampled from the population that are not known to be related and from which any pairs with a relationship above a low threshold have been removed, so as to minimise the chance of shared environment. Such an analysis is expected to give a lower estimate of heritability than the within-family analysis discussed here, however, because marker-associated effects in the population can be missed through incomplete linkage disequilibrium, especially when traits genes have low minor allele frequencies, as indeed seems to be the case [14].

A ‘back of the envelope’ calculation allows a simple comparison of the sampling errors of estimates of additive genetic variance from within families utilising variation in relationship, ${\hat{\sigma }}_{\mathrm{Aw}}^{2}$, and from between families using ANOVA, ${\hat{\sigma }}_{\mathrm{Ab}}^{2}$ (Appendix 3). Provided the families are not small, $\text{var}\left({\hat{\sigma }}_{\mathrm{Aw}}^{2}\right)/\text{var}\left({\hat{\sigma }}_{\mathrm{Ab}}^{2}\right)\approx \left(A^{2}/\sigma_{R}^{2}\right)/{\left[1+\mathrm{nA}\sigma_{A}^{2}/\sigma_{W}^{2}\right]}^{2}$. With use of half-sib families (*A* = 1/4) to eliminate maternal effects in the between-family estimate, for a genome of ‘human’ length, $\left(A^{2}/\sigma_{R}^{2}\right)$ = (0.25/0.028)^2^ ~ 80. Assuming the heritability is 1/3, such that $A\sigma_{A}^{2}=\frac{1}{5}\sigma_{W}^{2}$, the ratio of variances is approximately 80/(1 + *n*/5)^2^, equalling 1.0 when *n ~* 40. This implies that, with half-sib families of size 40, a similar amount of information would be obtained from within- and between-family data. With fewer larger families, the estimate from within-family information would have the lower standard error. Furthermore, because the within- and between-family estimates use the data in a different way they are, presumably, uncorrelated and so they can be simply combined. However, estimates from both sources may be biased to different extents by common environment, dominance, epistasis, etc., so specific applications require specific consideration.

There are other aspects that could be examined. For example, additive and within-family genetic covariances and correlations among traits can be estimated from a multi-trait analysis with the same data structure. Clearly the magnitude of their sampling errors is structured similarly to those of the corresponding variances of the individual traits. Estimation of variation due to any individual autosome can be achieved by fitting just the relationship on this chromosome, and similarly for the sex chromosome [6]. The variance of the corresponding relationships is then much higher and depends on the length of the chromosome, decreasing roughly in proportion to its length. Although $\text{var}\left({\hat{\sigma }}_{A}^{2}\right)$ per chromosome is then much smaller, the coefficient of variation of its estimate may be similar to that for the whole genome under the simplest assumption that the contribution by any chromosome to $\sigma_{A}^{2}$ is roughly proportional to its length.

A problem specific to the within-family approach is the high degree of confounding between additive and dominance effects in full-sib families (albeit there is also complete confounding in a between full-sib family analysis). This is not resolved by estimating $\sigma_{A}^{2}$ separately from maternal and paternal sharing, since the dominance coefficient is the correlated intersection of these. The point is that, while maternal genomic similarity appears to include only the additive component because only one sire is involved, interactions between sire and dam effects, i.e. dominance, are included. Half-sib families with multiple dams per sire or a cross classified structure are needed, similar to when between-family correlations are used for estimation.

If, for example, a number of males and females are put together for mating in a single environment, then the pedigree can be obtained from genetic markers. Hence, paternal half-sibs, maternal half-sibs and full-sibs can be distinguished and the between-family covariance can be used. Additional information from within-family segregation could be identified via the markers, but this would likely contribute little. For example, in a pen comprising such a diallel structure, the variation in pedigree relationships (*A* = 0, ¼ or ½) is likely to be much larger than the variation in realised relationships among pairs with the same pedigree relationship.

Epistatic variance provides other associated difficulties of potential confounding and estimation. On a whole-genome basis, the relevant coefficient for the additive × additive variance component is the square of the relationship, which is highly correlated with the additive coefficient. Thus, similar to the analyses between families, obtaining a satisfactory partition between additive and additive × additive or higher order components is probably not feasible. A further problem is potential bias due to epistatic effects in the estimation of additive (e.g. from additive × additive effects) and dominance variance. Although the expected probability that sibs share alleles at pairs of genomic sites is small for the genome as a whole, it is much higher for nearby sites. Thus, if epistatic effects are substantial and predominately cis-acting, this bias could be important. To partially address this, Visscher et al. [6] fitted the mean relationship for each chromosome in a multiple regression model for human height. The variance removed by fitting variation in relationships for each chromosome was essentially the same whether chromosomes were fitted independently or in a joint analysis, indicating little or no interaction between regions on different chromosomes. Extending this more generally needs genomic regions to be defined such that joint identity by descent can be computed.

Within-family analysis, particularly when families are large, has attractive features because. it avoids bias due to common environment effects, but it introduces other potential confounding effects, as noted above. It also requires much genotyping and associated costs. Although in a breeding context this type of information may be available when collecting data to implement genomic prediction and subsequent selection, estimates of the variance components may not in themselves have value beyond what is obtained from the marker trait associations. But this is something to think about.

## Appendix 1: Derivation of the sampling variance for the additive model

For the REML analysis, the information matrix **S**, which in turn yields the sampling variances based on **S**^-1^ for the estimates of $\sigma_{A}^{2}$ and $\sigma_{W}^{2}$ for each family, is defined by Lynch and Walsh (see page 791 in [2]):

(A1) $$S=\frac{1}{2}\left(\begin{array}{cc} \mathrm{tr}\left(\text{PRPR}\right) & \mathrm{tr}(\text{PRP}\\ \mathrm{tr}\left(\text{PRP}\right) & \mathrm{tr}\left(\mathrm{PP}\right) \end{array}\right),$$

where tr denotes the trace operator. Matrix **P** = **K**’**(KVK**’)^-1^**K** and **K**_(*n* – 1) x *n*_ defines contrasts such that **KX** = **0**, where **X** is the design matrix and, since family members are contemporaneous in the same environment, **X** is a unit vector. The Helmert contrasts are suitable for **K**: for *i* = 1, …, *n* – 1: *k*_*ij*_ = [(*i*(*i +* 1)]^-1/2^, *j* ≤ *i*; *k*_*i* ,*i* + 1_ = -[(*i*/(*i +* 1)]^1/2^ and *k*_*ij*_ = 0, *j > i* + 1. Note that **KK***’* = **I**_**(** *n -* 1)×(*n* - 1)_ and **K***’***K** = **I**_*n* × *n*_**-**$\frac{1}{n}$**J**_*n*× *n*_, where all elements of **J** equal 1, and (**K***’***K)**^**2**^ **= K***’***K**.

The expected information using the Taylor series expansion has terms of the following form:

$$\begin{array}{ll} E\left(\text{PRPR}\right)= & {\text{PRPR}}_{|R=0}+\sum_{i\leq j}\partial \left(\text{PRPR}\right)/{\partial r}_{\mathrm{ij}|R=0}E\left(r_{\mathrm{ij}}\right)\\ & +\frac{1}{2}\sum_{i\leq j}\sum_{k\leq l}\partial^{2}\left(\text{PRPR}\right)/{\partial r}_{\mathrm{ij}}{\partial r}_{\mathrm{kl}|R=0}E\left(r_{\mathrm{ij}}r_{\mathrm{kl}}\right)+\ldots \end{array}$$

We note that E(*r*_*ij*_) = 0 and, assuming independent Mendelian segregation to each offspring, E(*r*_*ij*_*r*_*kl*_) = 0, *i ≠ k* and/or *j ≠ l* and E(*r*_*ij*_)^2^ = $\sigma_{R}^{2}$, where $\sigma_{R}^{2}$ is the variance in relationship. Differentiating

(A2) $$\begin{array}{ll} \frac{\partial \left(\text{PRPR}\right)}{{\partial r}_{\mathrm{ij}}}= & \frac{\partial P}{{\partial r}_{\mathrm{ij}}}\text{RPR}+P\frac{\partial R}{{\partial r}_{\mathrm{ij}}}\mathrm{PR}+\mathrm{PR}\frac{\partial P}{{\partial r}_{\mathrm{ij}}}R\\ & +\text{RPR}\frac{\partial R}{{\partial r}_{\mathrm{ij}}}, \end{array}$$

and when evaluated as **R** → **0**, all terms in (A2) become zero. Furthermore, differentiating (A2) to obtain the second derivative, all remaining terms in **R** are also zero; and as **R** is linear in *r*_*ij*_, ∂^**2**^**R/**∂*r*_*ij*_∂*r*_*kl*_ = **0**. Finally, as E(*r*_*ij*_*r*_*kl*_) = 0 unless *i = k* and *j = l*, E(**PRPR**) reduces to

$$E\left(\text{PRPR}\right)\approx \frac{1}{2}\sum_{i<j}(P\frac{\partial R}{{\partial r}_{\mathrm{ij}}}P\frac{\partial R}{{\partial r}_{\mathrm{ij}}}+\frac{\partial R}{{\partial r}_{\mathrm{ij}}}P\frac{\partial R}{{\partial r}_{\mathrm{ij}}}P)\sigma_{R}^{2}.$$

Let ∂**R/***r*_*ij*_ = **X**_*ij*_, with elements *x*_*ij*_ **=** *x*_*ji*_ = 1 and 0 otherwise; so taking **R** → **0**,

(A3) $$E\left(\text{PRPR}\right)\approx \frac{1}{2}\sum_{i<j}\left({\mathrm{PX}}_{\mathrm{ij}}{\mathrm{PX}}_{\mathrm{ij}}+X_{\mathrm{ij}}{\mathrm{PX}}_{\mathrm{ij}}P\right)\sigma_{R}^{2}$$

As **R** → **0**, **V** → **P** = **K**’**(KVK**’)^-1^**K** → (**I** – $\frac{1}{n}$**J**)/$\sigma_{W}^{2}$. Defining further matrices, **Y**_*ij*_ where *y*_*ii*_ = *y*_*jj*_ = 1 and 0 otherwise, and **W**_*ij*_ where *w*_*ik*_ = *w*_*jk*_ *=* 1, *k* = 1, …, *n*, and 0 otherwise, we have **X**_***ij***_**X**_***ij***_ = **Y**_*ij*_, **JX**_*ij*_ = **JY**_*ij*_ = **W**_***ij***_, **W**_*ij*_**W**_*ij*_ **=** 2**W**_*ij*_, and tr(**X**_*ij*_) = 0, tr(**Y**_*ij*_) = tr(**W**_*ij*_) = 2. As the trace operator is commutative, it follows that by summing over the *n*(*n* – 1)/2 off diagonal elements in (A3), all having the same expectation,

(A4) $$\begin{array}{ll} E\left[\mathrm{tr}\left(\text{PRPR}\right)\right]\; & \approx \frac{1}{2}n\left(n-1\right)\mathrm{tr}\left[\left(I–J/n)X_{\mathrm{ij}}\left(I-J/n\right)X_{\mathrm{ij}}\right)\right]\sigma_{R}^{2}/\sigma_{W}^{4}\\ & \approx \frac{1}{2}n\left(n-1\right)\mathrm{tr}\left(Y_{\mathrm{ij}}–2W_{\mathrm{ij}}/n+2W_{\mathrm{ij}}/n^{2}\right)\sigma_{R}^{2}/\sigma_{W}^{4}\\ & \approx n\left(n-1\right)\left(1–2/n+2/n^{2}\right)\sigma_{R}^{2}/\sigma_{W}^{4}=\left(n-1\right)m\sigma_{R}^{2}/\sigma_{W}^{4} \end{array}$$

where *m* = *n*(1 – 2/*n* + 2/*n*^2^).

We give less detail for other terms in the information matrix.

$$\frac{\partial \left(\text{PRP}\right)}{{\partial r}_{\mathrm{ij}}}=\frac{\partial P}{{\partial r}_{\mathrm{ij}}}\mathrm{RP}+P\frac{\partial R}{{\partial r}_{\mathrm{ij}}}P+\mathrm{PR}\frac{\partial P}{{\partial r}_{\mathrm{ij}}}.$$

Non-zero second derivatives must involve differentiation once of **P** and once of **R**. Hence

(A5) EPRP≈12∑i<j(2∂P∂rij∂R∂rijP+2P∂R∂rij∂P∂rij)σR2∂P∂rij=-K'KVK'-1K∂V∂rijK'KVK'-1Kand,asR→0,∂P∂rij→-I-1nJXij(I-1nJ)σA2/σW6,soEPRP≈12∑i<j-4I-1nJXijI-1nJXijI-1nJσR2σA2/σW6

As the trace is commutative and **I –**$\frac{1}{n}$**J** is idempotent, putting the last such matrix in (A5) first, we see that:

$$\begin{array}{l} E\left[\mathrm{tr}\left(\mathrm{PRP}\right)\right]\approx -2n\left(n-1\right)\left(1–2/n+2/n^{2}\right)\sigma_{A}^{2}\sigma_{R}^{2}/\sigma_{W}^{6}\\ \;=-2\left(n-1\right)m\;\sigma_{A}^{2}\sigma_{R}^{2}/\sigma_{W}^{6}. \end{array}$$

When **R** = **0**, **P** = (1 – 1/*n*)/$\sigma_{W}^{2}$ and tr(**PP)** = (*n* – 1)/$\sigma_{W}^{4}$. Now considering the terms in *r*_*ij*_,

(A6) $$\frac{\partial^{2}\left(\mathrm{PP}\right)}{\partial r_{\mathrm{ij}}^{2}}=\frac{\partial^{2}P}{\partial r_{\mathrm{ij}}^{2}}P+2\frac{\partial P}{\partial r_{\mathrm{ij}}}\frac{\partial P}{\partial r_{\mathrm{ij}}}+P\frac{\partial^{2}P}{\partial r_{{}_{\mathrm{ij}}}^{2}}$$

with additional terms that become 0 as **R** → **0**.

In (A6) ∂P∂rij=-K'KVK'-1K∂V∂rijK'KVK'-1K

∂2P∂rij2=2K'KVK'-1K∂V∂rijK'KVK'-1K∂V∂rijK'KVK'-1K

And hence, using the commutative property,

tr∂2PP∂rij2=6trK'KVK'-1K∂V∂rijK'KVK'-1K∂V∂rijK'KVK'-1K=6tr(I-1nJXij(I-1nJ)Xij(I-1nJ))σA4/σW8.

Therefore, using previous results,

$$E\left[\mathrm{tr}\left(\mathrm{PP}\right)\right]\approx \left(n-1\right)/\sigma_{W}^{4}+3\mathrm{nm}\;\sigma_{R}^{2}\sigma_{A}^{4}/\sigma_{W}^{8}$$

thus completing the derivation of the information matrix in Equation (1) of the main text.

## Appendix 2: Fitting additive and dominance variances

Let $V=I\sigma_{w}^{2}+R\sigma_{A}^{2}+Q\sigma_{D}^{2}$ of dimension *n* × *n*, where, for full sib families, $\sigma_{W}^{2}=\sigma_{E}^{2}+\frac{1}{2}\sigma_{A}^{2}+\frac{3}{4}\sigma_{D}^{2}$. Additive and dominance effects of the loci are assumed to be uncorrelated. Let **Q** with elements *q*_*ij*_ define the departure of the realised dominance correlation of full sibs from the expected ¼, and let $\sigma_{Q}^{2}$ denote var(*q*_*ij*_) and similarly *cov*_RQ_ denote cov(*r*_*ij*_, *q*_*ij*_). The information matrix is now [2]:

$$S=\frac{1}{2}\left(\begin{array}{ccc} \mathrm{tr}\left(\text{PRPR}\right) & \mathrm{tr}\left(\text{PRPQ}\right) & \mathrm{tr}(\text{PRP}\\ \mathrm{tr}\left(\text{PRPQ}\right) & \mathrm{tr}\left(\text{PQPQ}\right) & \mathrm{tr}\left(\text{PQP}\right)\\ \mathrm{tr}\left(\text{PRP}\right) & \mathrm{tr}\left(\text{PQP}\right) & \mathrm{tr}\left(\mathrm{PP}\right) \end{array}\right).$$

The term E[tr(**PRPR)]** ≈ (*n -*1)*m*$\sigma_{R}^{2}/\sigma_{W}^{4}$ is unchanged from the additive case and, by symmetry,

$$\begin{array}{l} E\left[\mathrm{tr}\left(\text{PQPQ}\right)\right]\approx \left(n-1\right){m\sigma }_{Q}^{2}/\sigma_{W}^{4}\;\text{and}\\ E\left[\mathrm{tr}\left(\text{PRPQ}\right)\right]\approx \left(n-1\right){\text{mcov}}_{\mathrm{RQ}}/\sigma_{W}^{4}. \end{array}$$

The derivative of the term **PRP** with respect to *r*_*ij*_ remains

$$\frac{\partial \left(\text{PRP}\right)}{{\partial r}_{\mathrm{ij}}}=\frac{\partial P}{{\partial r}_{\mathrm{ij}}}\mathrm{RP}+P\frac{\partial R}{{\partial r}_{\mathrm{ij}}}P+\mathrm{PR}\frac{\partial P}{{\partial r}_{\mathrm{ij}}}\text{,}$$

and the expectation of its second derivative with respect to *r*_*ij*_ is unchanged. However, now taking the second derivative with respect to *q*_*ij*_, we obtain additional terms with non zero expectation,

$$\frac{\partial^{2}\left(\text{PRP}\right)}{{\partial r}_{\mathrm{ij}}\partial q_{\mathrm{ij}}}=\frac{\partial P}{\partial q_{\mathrm{ij}}}\frac{\partial R}{{\partial r}_{\mathrm{ij}}}P+P\frac{\partial R}{{\partial r}_{\mathrm{ij}}}\frac{\partial P}{\partial q_{\mathrm{ij}}}.$$

$$\text{Hence}\;E\left[\mathrm{tr}\left(\text{PRP}\right)\right]\approx -2\left(n-1\right)m\left(\sigma_{R}^{2}\sigma_{A}^{2}+{\mathrm{cov}}_{\mathrm{RQ}}\sigma_{D}^{2}\right)/\sigma_{W}^{4},$$

and similarly

E[tr(**PQP**)] ≈ $-2\left(n-1\right)m\left({\mathrm{cov}}_{\mathrm{RQ}}\sigma_{A}^{2}+\sigma_{Q}^{2}\sigma_{D}^{2}\right)/\sigma_{W}^{4}$. The term E[tr(**PP**)] is non-zero when differentiated twice with respect to *r*_*ij*_ and to *q*_*ij*_ and once each with both variables. Hence

$$\begin{array}{ll} E\left[\mathrm{tr}\left(\mathrm{PP}\right)\right]\approx & \left(n-1\right)/\sigma_{W}^{4}\\ & +3\left(n-1\right)m\left(\sigma_{R}^{2}\sigma_{A}^{4}+2\mathrm{co}v_{\mathrm{RQ}}\sigma_{A}^{2}\sigma_{D}^{2}+\sigma_{Q}^{2}\sigma_{D}^{4}\right)/\sigma_{W}^{8}. \end{array}$$

The information matrix for a single family is therefore

$$\begin{array}{ll} S= & \left(\frac{n-1}{2\sigma_{W}^{4}}\right)\\ & \times \left(\begin{array}{ccc} m\sigma_{R}^{2} & m{\text{cov}}_{\mathrm{RQ}} & -2m\left(\sigma_{R}^{2}\sigma_{A}^{2}+\sigma_{D}^{2}\mathrm{co}v_{\mathrm{RQ}}\right)/\sigma_{W}^{2}\\ & m\sigma_{Q}^{2} & -2m\left(\mathrm{co}v_{\mathrm{RQ}}\sigma_{A}^{2}+\sigma_{Q}^{2}\sigma_{D}^{2}\right)/\sigma_{W}^{2}\\ \text{symm} & & 1+3m\left(\sigma_{R}^{2}\sigma_{A}^{4}+2\mathrm{co}v_{\mathrm{RQ}}\sigma_{A}^{2}\sigma_{D}^{2}+\sigma_{Q}^{2}\sigma_{D}^{4}\right)/\sigma_{W}^{4} \end{array}\right). \end{array}$$

These equations apply to estimates of ${\overset{⌢}{\sigma }}_{A}^{2}$ , ${\hat{\sigma }}_{D}^{2}$ and ${\hat{\sigma }}_{W}^{2}$. For full sib families, the estimate of the error variance would be ${\overset{⌢}{\sigma }}_{E}^{2}$=${\overset{⌢}{\sigma }}_{W}^{2}$-$\frac{1}{2}{\overset{⌢}{\sigma }}_{A}^{2}$-$\frac{3}{4}{\overset{⌢}{\sigma }}_{D}^{2}$, and its sampling error computed accordingly from **S**^-1^.

As noted in the main text, *cov*_RQ_ =$\sigma_{R}^{2}$, so **S** simplifies to

$$S=\frac{n-1}{{2\sigma }_{W}^{4}}\left(\begin{array}{ccc} {m\sigma }_{R}^{2} & {m\sigma }_{R}^{2} & {-2m\sigma }_{R}^{2}\left(\sigma_{A}^{2}+\sigma_{D}^{2}\right)/\sigma_{W}^{2}\\ & {m\sigma }_{Q}^{2} & -2m\left(\sigma_{R}^{2}\sigma_{A}^{2}+\sigma_{Q}^{2}\sigma_{D}^{2}\right)/\sigma_{W}^{2}\\ \mathrm{symm} & & 1+3m[\sigma_{R}^{2}\left(\sigma_{A}^{2}+2\sigma_{D}^{2}\right)\sigma_{A}^{2}+\sigma_{Q}^{2}\sigma_{D}^{4}]/\sigma_{W}^{4} \end{array}\right).$$

However, as $\sigma_{R}^{2}$ and $\sigma_{Q}^{2}$ have similar magnitude, **S** is almost singular and thus the genotypic variance cannot be partitioned into additive and dominance components unless the dataset is very large.

## Appendix 3: Comparison of between and within family estimators

Let us assume a balanced one-way ANOVA (which is also REML if there are no unbalanced fixed effects) is used to estimate $\sigma_{A}^{2}$, i.e. ${\hat{\sigma }}_{\mathrm{Ab}}^{2}$ = (*MSB – MSW*)/(*nA*) where *MSB* and *MSW* are the mean squares and *A* is the pedigree relationship (½ or ¼). It is assumed that there is no environmental correlation among sibs. Hence, with *f* families each of size *n*, $\text{var}\left(\text{MSB}\right)=2{\left[\sigma_{W}^{2}+\left(n-1\right)A\sigma_{A}^{2}\right]}^{2}/\left(f-1\right)$, $\text{var}\left(\text{MSW}\right)=2\sigma_{W}^{4}/\left[f\left(n-1\right)\right]$ and, as these are uncorrelated,

$$\text{var}\left({\hat{\sigma }}_{\mathrm{Ab}}^{2}\right)=\frac{2\sigma_{W}^{4}}{{\left(\mathrm{nA}\right)}^{2}}\left(\frac{{\left[1+\left(n-1\right)\left(A\sigma_{A}^{2}/\sigma_{W}^{2}\right)\right]}^{2}}{f-1}+\frac{1}{f\left(n-1\right)}\right).$$

For the within-family estimates, $\text{var}\left({\hat{\sigma }}_{\mathrm{Aw}}^{2}\right)$ is given by (3). Further simplification requires making some assumptions about numbers and size of families. As a first approximation, assume neither is small, so

$$\begin{array}{l} \text{var}\left({\hat{\sigma }}_{\mathrm{Ab}}^{2}\right)\approx \;\frac{{2\sigma_{W}^{4}[1+\mathrm{nA}\sigma_{A}^{2}/\sigma_{W}^{2})]}^{2}}{fn^{2}A^{2}},\;\text{var}\left({\hat{\sigma }}_{\mathrm{Aw}}^{2}\right)\approx \frac{2\sigma_{W}^{4}}{fn^{2}\sigma_{R}^{2}}\\ \;\text{and}\;\frac{\text{var}\left({\hat{\sigma }}_{\mathrm{Aw}}^{2}\right)}{\text{var}\left({\hat{\sigma }}_{\mathrm{Ab}}^{2}\right)}\approx \frac{A^{2}/\sigma_{R}^{2}}{{\left[1+\mathrm{nA}\sigma_{A}^{2}/\sigma_{W}^{2}\right]}^{2}}. \end{array}$$

## Competing interests

The author declares no competing interests.

## Author’s contributions

WGH proposed, executed and reported the study.
