# Erratum to: On estimation of genetic variance within families using genome-wide identity-by-descent sharing

**DOI:** 10.1186/s12711-014-0062-8

**Published:** 2014-10-15

**Authors:** William G Hill

**Affiliations:** Institute of Evolutionary Biology, School of Biological Sciences, University of Edinburgh, West Mains Road, Edinburgh, EH9 3JT UK

## Erratum

After publication of this work [1], regrettably I noted that there was an error in the examples given in Table 1, which was added when the paper was under revision and inadequately checked. A programming error led to incorrect calculations of within-family variances for full-sib families. Formulae and conclusions are unchanged.

**Table 1:** **Comparison of var(** ${\widehat{\sigma}}_{\mathrm{A}}^2$ ) predicted from the information matrix directly and from the Taylor series approximation*

**Family**	**HS**			**FS**			**FS**			**FS**			**FS**		
*h* ^2^	0.5			0.25			0.5			0.75			0.04		
chr	22			22			22			22			1		
*n*	5	15	25	5	15	25	5	15	25	5	15	25	5	15	25
Eq (A1)	174	12.2	4.15	**82.2**	**5.82**	**1.97**	**60.7**	**4.39**	**1.52**	**42.2**	**3.09**	**1.09**	**4.69**	**0.347**	**0.125**
Eq (1)	182	11.8	3.88	**88.2**	**5.69**	**1.90**	**65.2**	**4.32**	**1.47**	**46.1**	**3.23**	**1.16**	**5.16**	**0.324**	**0.107**
Eq (3)	182	11.7	3.80	**88.1**	**5.69**	**1.88**	**64.7**	**4.18**	**1.47**	**44.9**	**2.90**	**0.96**	**5.15**	**0.323**	**0.107**

*Predictions were obtained directly by inverting the realised information matrix (eq A1) obtained from sampling relationships, and from the Taylor series approximation eq. (1) using the variance of relationships directly. Variances, assuming ${\sigma}_{\mathrm{p}}^2=1$, were computed by averaging information over samples of 100 families, but are expressed for a single family, so for *f* families var(${\widehat{\sigma}}_{\mathrm{A}}^2$) should be divided by *f*. Predictions using the simplification eq. (3) are shown similarly; results are for half (HS) and full (FS) sib families; *h* ^2^ is the proportion of variance contributed by the fitted chromosomes; chr is the number of chromosomes; chr = 22 denotes the whole genome; chr = 1 denotes a single chromosome.

The correct Table 1 is included here. Table title, headings and footnote are unchanged. Results are changed in columns 4 through 15 and indicated in bold characters.
